# Sustained Insulin Independence Following Lifestyle Intervention in a 78-Year-Old Patient With Long-Standing Type 2 Diabetes: A Six-Year Follow-Up Case Report

**DOI:** 10.7759/cureus.107273

**Published:** 2026-04-18

**Authors:** Pramod Tripathi, Nidhi S Kadam, Thejas Kathrikolly, Malhar Ganla

**Affiliations:** 1 Department of Research, Freedom From Diabetes Research Foundation, Pune, IND; 2 Department of Medicine, Freedom From Diabetes Clinic, Pune, IND; 3 Department of Management and Exercise Science, Freedom From Diabetes Clinic, Pune, IND

**Keywords:** geriatric diabetes, insulin deprescribing, insulin discontinuation, lifestyle intervention, type 2 diabetes

## Abstract

Insulin dependence in elderly patients with long-standing type 2 diabetes (T2D) is common due to progressive β-cell decline, and insulin withdrawal is rarely considered. Evidence on sustained insulin discontinuation and long-term glycemic stability following intensive lifestyle interventions (ILIs) in elderly patients with long-standing T2D is limited.

This case report describes a 78-year-old man with a 40-year history of T2D and 31 years of insulin therapy who achieved insulin independence after a structured ILI program. The multidisciplinary protocol incorporated a whole-food, plant-based diet, intermittent fasting, yoga, resistance training, and stress management, with medication deprescription guided by the Research Society for the Study of Diabetes in India-Endocrine Society of India (RSSDI-ESI) Therapeutic Wheel, monitored through daily remote tracking of glucose levels. Insulin was successfully tapered and discontinued within the first month of enrolment. At 10 months, weight decreased from 71.9 to 62.5 kg, body mass index (BMI) from 24.9 to 21.6 kg/m², and glycated hemoglobin (HbA1c) from 7.0% to 6.6%. Lipids and inflammation improved (triglycerides, 59 to 41 mg/dL; high-density lipoprotein cholesterol (HDL-C), 53 to 62 mg/dL; high-sensitivity C-reactive protein (hs-CRP), 0.7 to 0.3 mg/L), and renal function remained stable. At the 77-month follow-up, the patient maintained an insulin-free status on minimal oral therapy (gliclazide), with stable weight (63.5 kg) and HbA1c (6.6%). Fasting insulin (4.2 μU/mL) and C-peptide (1.36 ng/mL) levels confirmed preserved endogenous insulin secretion.

This case illustrates that even elderly patients with very long-standing T2D may achieve sustained insulin withdrawal in the context of comprehensive ILI, underscoring the importance of integrating lifestyle medicine into diabetes care to reduce polypharmacy and enhance long-term outcomes.

## Introduction

Type 2 diabetes (T2D) affects 537 million adults globally, with a particularly high prevalence among older adults, affecting approximately 33% of those aged ≥65 years [1]. Geriatric patients with long-standing diabetes frequently require insulin therapy owing to progressive β-cell decline [2], and nearly one-fifth of those aged ≥75 years are insulin-dependent, particularly those in poor health [3]. However, insulin use in this population carries significant challenges: the risk of severe hypoglycemia is fourfold higher than that in younger patients [3], while age-related renal dysfunction, irregular meal patterns, and polypharmacy further complicate management [4]. Despite these well-recognized concerns, insulin deprescribing in older adults remains an underexplored clinical practice.

Intensive lifestyle interventions (ILIs), comprising structured dietary modifications, physical activity, and behavioral support, have emerged as promising approaches for T2D management. Landmark trials, including the Diabetes Remission Clinical Trial (DiRECT) and Diabetes Intervention Accentuating Diet and Enhancing Metabolism (DIADEM) studies, have demonstrated that substantial weight loss can induce diabetes remission, particularly in individuals with a shorter disease duration [5,6]. Within this framework, whole-food, plant-based dietary patterns have been associated with improved glycemic control, and regular physical activity has been linked to enhanced insulin sensitivity and glucose utilization [7,8]. However, evidence supporting such approaches in elderly individuals with long-standing, insulin-dependent T2D, where disease chronicity and β-cell exhaustion are more advanced, is limited.

In this context, we present the case of a 78-year-old man with a 40-year history of T2D and 31 years of insulin use who achieved sustained insulin discontinuation following a structured ILI, maintaining glycemic control for six years with minimal oral therapy (gliclazide). This case highlights the potential reversibility of insulin dependence, even in advanced disease stages, and underscores the possible role of residual β-cell function and improved insulin sensitivity in achieving sustained glycemic stability. This further illustrates the potential of lifestyle medicine to reduce the treatment burden and support the safe de-escalation of therapy in elderly patients who are traditionally considered permanently insulin-dependent.

## Case presentation

A 78-year-old man was diagnosed with T2D in April 1979 at the age of 34 years during routine screening. Initially managed with oral hypoglycemic agents, including metformin, for nine years, insulin therapy was initiated in April 1988 due to progressive hyperglycemia and continued for 31 years. Metformin was continued alongside insulin therapy to improve insulin sensitivity and reduce total insulin requirements, which is consistent with standard management practices for T2D. The diagnosis of T2D was based on adult-onset hyperglycemia at the age of 34 years, absence of ketoacidosis at presentation, and lack of clinical features suggestive of type 1 diabetes. His treatment pattern at presentation was consistent with the progressive β-cell dysfunction typically observed in patients with T2D. The patient had no history of autoimmune disease or unexplained weight loss at the time of diagnosis. At ILI program enrolment in April 2019, he weighed 71.9 kg (body mass index (BMI) 24.9 kg/m²), with a waist circumference of 94 cm. Bioelectrical impedance analysis (Karada Scan; Omron Healthcare Co., Ltd., Kyoto, Japan) revealed sarcopenic obesity (defined as the co-existence of excess adiposity and low muscle mass/function) [9]: 31.1% total body fat, 21.1% subcutaneous fat, and 26.6% skeletal muscle mass, with a visceral fat level of 13.5. He followed a vegetarian diet and regularly practiced yoga and walking exercises. His medical history included hypertension and hypothyroidism (diagnosed in 1987), myocardial infarction with coronary angioplasty (1987), coronary artery bypass graft surgery (2011), hypercholesterolemia, hernia repair (2007), and surgery for prostate enlargement (2009). At enrolment, the medications included regular insulin (8 units daily), human insulin 50/50 (32 units daily), metformin (750 mg daily) for glycemic management, bisoprolol fumarate, aspirin for cardiovascular health, and levothyroxine sodium for hypothyroidism management.

Intervention

At enrolment, laboratory investigations indicated moderate glycemic and metabolic dysfunction. Despite dual therapy with insulin and oral hypoglycemic agents, the glycated hemoglobin (HbA1c) and fasting glucose levels were 7.0% and 108.3 mg/dL, respectively. The Quantitative Insulin Sensitivity Check Index (QUICKI) score was 0.31, reflecting impaired insulin sensitivity [10]. The lipid profile showed high-density lipoprotein cholesterol (HDL-C) of 53 mg/dL, low-density lipoprotein cholesterol (LDL-C) of 67 mg/dL, and triglyceride levels of 59 mg/dL. Renal function was preserved, with an estimated glomerular filtration rate (eGFR) of 86 mL/min/1.73 m² and a serum creatinine level of 0.87 mg/dL. Other laboratory parameters included vitamin B12 (828 pg/mL), vitamin D (24.5 ng/mL), thyroid-stimulating hormone (1.63 μIU/mL), and hemoglobin levels (12.4 g/dL). The high-sensitivity C-reactive protein (hs-CRP) level was low, at 0.7 mg/L (normal <1 mg/L), indicating no systemic inflammation. Uric acid was slightly elevated at 6.81 mg/dL but was within the typical male reference range of 3.4-7.0 mg/dL [11].

Intensive lifestyle intervention (ILI)

Following enrolment in April 2019, the patient received a structured, phase-wise plan comprising a whole-food, plant-based diet, physical activity, stress management, and medical supervision. A multidisciplinary team, comprising a physician, dietitian, physical therapist, and mentor (past participant), provided ongoing support. Blood glucose levels were tracked daily using a mobile application [12], allowing real-time monitoring and physician-guided medication adjustments.

Medication withdrawal followed the Research Society for the Study of Diabetes in India-Endocrine Society of India (RSSDI-ESI) Therapeutic Wheel [13], which incorporates HbA1c, diabetes duration, weight, hypoglycemia risk, and comorbidities in treatment decision-making. In this framework, older age, risk of hypoglycemia, and achievement of glycemic targets supported insulin discontinuation, with the continuation of minimal oral therapy and lifestyle measures. This structured approach, coupled with continuous monitoring and clinical judgment, enabled safe deprescribing and maintained glycemic parameters within an acceptable range, with expected day-to-day physiological variability during the medication recalibration phase.

For the first six months, the patient attended in-person clinic visits, including body composition assessment using the Karada Scan device (a bioelectrical impedance analysis-based body composition analyzer providing measurements of body weight, body fat percentage, visceral fat, skeletal muscle mass, and BMI). From February 2020, due to COVID-19 restrictions, follow-ups transitioned to teleconsultations supported by a mobile application. Although body composition data could not be captured, other physical measurements were self-reported by the patient and verified by the physician, ensuring data reliability.

Diet

The patient, with a baseline BMI of 24.9 kg/m², was prescribed a structured plant-based diet to stabilize glucose levels, improve satiety, and support metabolic health. Green smoothies (leafy greens and seasonal fruits) were consumed twice daily (500 mL/day) on an empty stomach [14]. Breakfast included 50% raw foods (salads and sprouts) and 50% cooked pulses or lentils but excluded cereals. All dairy products were eliminated, and green and black tea were permitted. Lunch and dinner comprised equal portions of a single grain, cooked vegetables, raw salad, and pulses/lentils. The evening intake included mixed seeds, soaked almonds, and walnuts. The macronutrient distribution targeted 65-70% carbohydrates, 10-15% protein, and 20-25% fat, prioritizing carbohydrate quality over restriction. The diet was adapted for hypothyroidism and cardiovascular risk by limiting goitrogenic and cholesterol-raising foods (e.g., soybeans, cruciferous vegetables, millet, peaches, and peanuts). Once the BMI was normalized, calorie intake was gradually increased (~2200 kcal/day) with energy-dense plant foods to support muscle building.

Exercise

The patient was prescribed a phased exercise protocol tailored to support metabolic recovery and muscle strengthening. The initial phase included warm-up routines [15], yoga-based exercises such as Super Brain Yoga [16] and chair Suryanamaskar, walking or walk-jog sessions, and anti-gravity exercises such as stair climbing. In the second phase, yoga-based cleansing practices including whole-system breathing, dhauti kriyas, and kapalbhati were introduced, alongside mild strength-building exercises with light weights and resistance bands, with a minimum of 45 minutes of daily activity. The third phase focused on building strength and stamina through a balanced weekly routine comprising three hours of resistance training, two hours of cardiovascular activity, and one hour of flexibility exercises.

Stress management

Psychological and behavioral support focused on stress alleviation through daily meditation, emphasizing mindfulness, deep breathing exercises, and positive affirmations, was provided. Regular journaling was encouraged for stress relief, self-reflection, and emotional processing.

Medical management

Daily glucose monitoring using a mobile application enabled proactive adjustment of the medication. Deprescribing followed the RSSDI-ESI Therapeutic Wheel, with close physician oversight to prevent hypoglycemia. Vitamin D and B12 supplementation were provided to maintain adequate levels.

There were no reported adherence-related difficulties throughout the intervention, potentially facilitated by the patient’s pre-existing vegetarian diet and active lifestyle, along with age- and fitness-appropriate tailoring of the regimen.

Outcome and follow-up

The patient showed a remarkable and sustained positive response to ILI over the 77-month follow-up period. The most critical early milestone was the complete discontinuation of insulin within the first month. By seven months, peak improvements in body composition were observed, followed by progressive stabilization of glycemic parameters between 10 and 19 months. The maximum reduction in HbA1c was recorded at 56 months, and the final assessment at 77 months confirmed the long-term sustainability of the insulin-free status with continued metabolic stability (Table 1).

**Table 1:** Changes in anthropometric and biochemical parameters during intervention and follow-up ^†^ With medication and insulin, subsequent values are with OHA; ^‡ ^April 30, 2019; * As of September 2025, the patient continued to receive 60 mg gliclazide twice daily at the latest follow-up; ⁑ Indices and classification systems (including the RSSDI-ESI Therapeutic Wheel) are publicly available and free for academic use; References [17-25] correspond to the reference ranges for the respective parameters provided in the table. M, months; BMI, body mass index; HbA1c, glycated hemoglobin; LDL-C, low-density lipoprotein cholesterol; HDL-C, high-density lipoprotein cholesterol; hs-CRP, high-sensitivity C-reactive protein; eGFR, estimated glomerular filtration rate; QUICKI, quantitative insulin-sensitivity check index; OHA, oral hypoglycemic agents; RSSDI-ESI, Research Society for the Study of Diabetes in India-Endocrine Society of India

Parameter	Reference range	Baseline^‡^	3 M	7 M	10 M	15 M	19 M	37 M	56 M	77 M*
Weight (kg)		71.9	63.4	63.9	62.5	61.5	62.5	-	-	63.5
BMI [17]^⁑^	18.5-22.9 (Asian population)	24.9	21.9	22.1	21.6	21.3	21.6	-	-	21.9
HbA1c% [18]	<5.7 (normal)	7.0^†^	7.0	6.9	6.6	6.5	6.5	6.5	6.1	6.6
Fasting blood glucose (mg/dL) [18]	70-99	108.3^†^	117.0	123.8	102.4	90.3	97.4	-	-	85.3
Fasting insulin (μU/mL) [19]	2-25	14.5^†^	4.8	12.9	9.2	4.9	11.6	-	-	4.2
QUICKI [10]^⁑^	~0.33-0.45	0.31	0.36	0.31	0.34	0.38	0.33	-	-	0.39
Total cholesterol (mg/dL) [20]	<200	121.0	124.0	-	-	123.0	121.4	-	-	146.0
Triglycerides (mg/dL) [20]	<150	59.0	50.0	-	-	33.0	35.9	-	-	41.0
LDL-C (mg/dL) [20]	<100	67.0	68.0	-	-	60.0	55.0	-	-	76.0
HDL-C (mg/dL) [20]	>40 (men)	53.0	44.0	-	-	52.0	59.7	-	-	62.0
hs-CRP (mg/L) [21]	<1.0	0.7	0.7	-	-	0.5	0.6	-	-	0.3
Serum creatinine (mg/dL) [22]	0.7-1.3	0.9	0.8	0.8	-	0.8	0.9	-	-	0.8
eGFR (mL/min/1.73 m²) [23]	≥90 (normal)	86	91	95	-	88	89	-	-	-
Vitamin B12 (pg/mL) [24]	200-900	828	349	-	-	315	471.2	-	-	266
Vitamin D (ng/mL) [25]	30-100	24.5	52.7	-	-	39.4	72.6	-	-	24.6

The most significant early outcome was the gradual tapering, with complete discontinuation of insulin therapy by the end of one month (May 1, 2019), which marked a 100% reduction in insulin requirements (complete discontinuation), a 9.4 kg (13.1%) weight loss by 10 months, and a reduction in visceral fat level from 13.5 to 9 (33.3%). The weight at the final follow-up (77 months) was 63.5 kg, representing a sustained reduction of 8.4 kg (11.7%) from baseline, confirming long-term maintenance of weight loss.

At 10 months, HbA1c reduced from baseline 7.0% to 6.6%, fasting blood glucose dropped from 108.3 mg/dL to 102.4 mg/dL, and fasting insulin declined substantially from 14.5 µU/mL to 9.2 µU/mL. This was accompanied by improved insulin sensitivity, as reflected by an increase in the QUICKI score from 0.31 to 0.34. At 19 months, although there was a modest decrease in the QUICKI to 0.33 (reference range > 0.34), it remained within the acceptable range for non-insulin-dependent diabetes patients, especially in the geriatric context.

At 77 months, there was a sustained improvement in glycemic control, with reductions in HbA1c and fasting glucose, along with marked improvements in fasting insulin and QUICKI. Additionally, HDL cholesterol levels improved, indicating favorable changes in the lipid profile (Table 2).

**Table 2:** Quantitative assessment of key clinical parameters HbA1c, glycated hemoglobin; QUICKI, quantitative insulin-sensitivity check index; HDL, high density lipoprotein

Parameter	Baseline	77 months	Percentage change
HbA1c	7.0%	6.6%	5.7% reduction
Fasting glucose	108.3 mg/dL	85.3 mg/dL	21.2% reduction
QUICKI	0.31	0.39	25.8% improvement
Fasting insulin	14.5 μU/mL	4.2 μU/mL	71.0% reduction
HDL cholesterol	53 mg/dL	62 mg/dL	17.0% improvement

**Figure 1:**
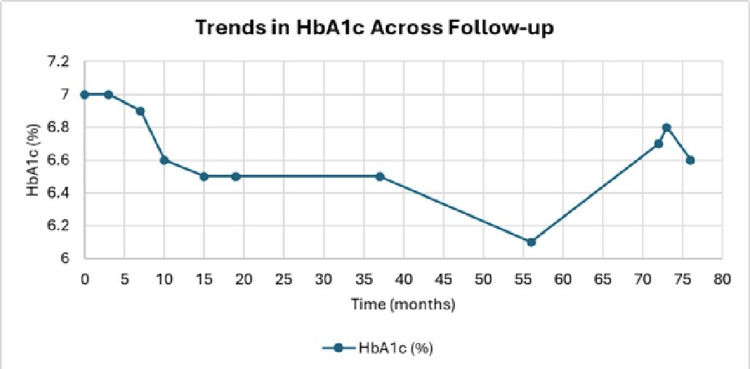
Trends in HbA1c across follow-up Baseline (0 month) HbA1c level with OHAs and insulin; later readings reflect “OHA only.” HbA1c, glycated hemoglobin; OHA, oral hypoglycemic agents

**Figure 2:**
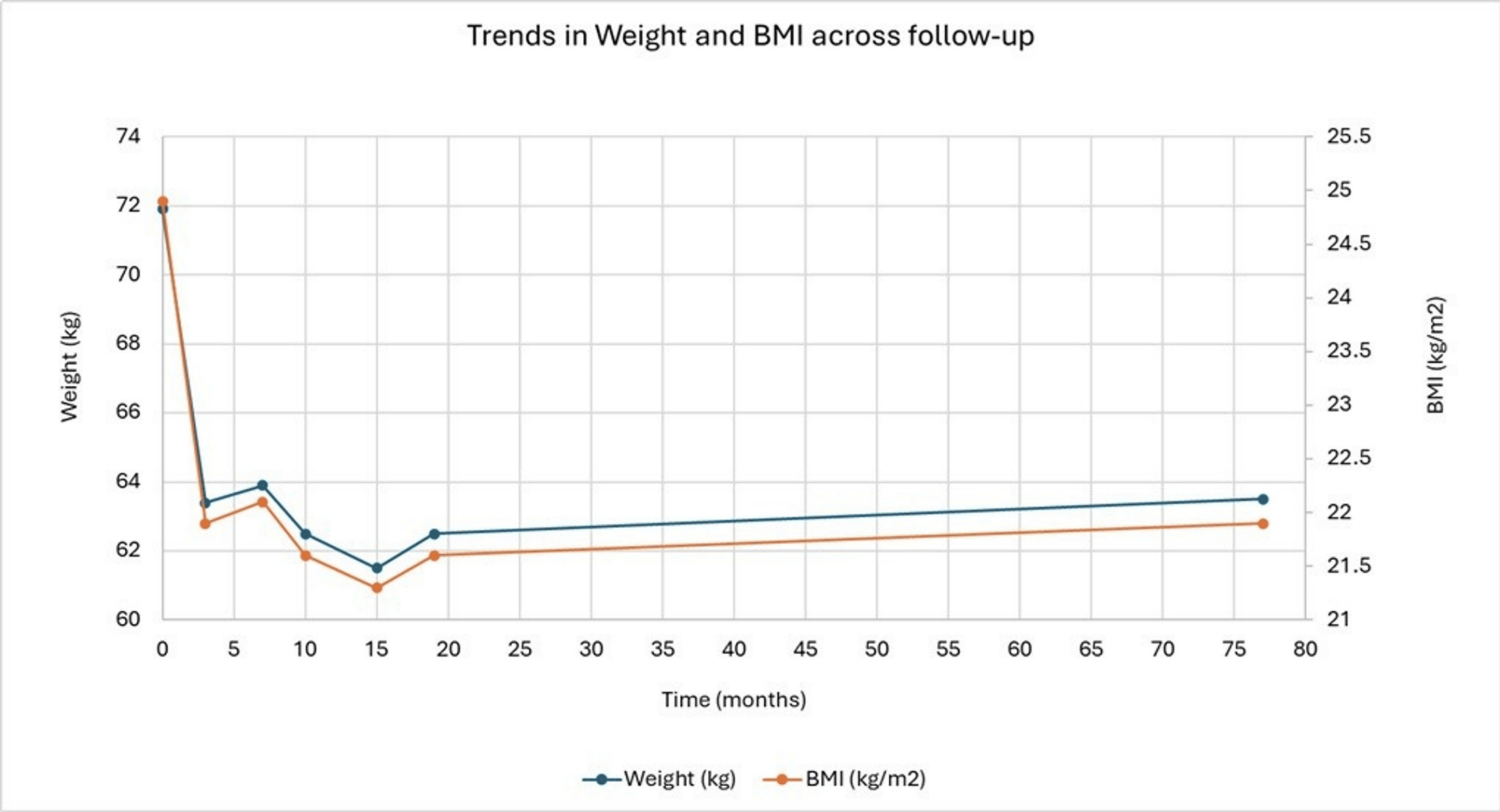
Trends in weight and BMI across follow-up BMI, body mass index

These biochemical improvements paralleled a steady reduction in weight and BMI, with a total weight loss of 9.4 kg and waist circumference improvement from 94 to 89 cm at the 10-month follow-up visit. Trends in clinical (HbA1c) and anthropometric (weight and BMI) parameters across the follow-up period are depicted in Figures 1-2, respectively. Renal function remained stable throughout the follow-up period, with eGFR ranging between 88 and 89 mL/min/1.73 m² at the last available measurements (15 and 19 months).

Furthermore, HDL increased from 53.0 to 62.0 mg/dL, and LDL cholesterol and triglyceride levels remained within normal limits (with lipid-lowering medication). Renal function remained stable, with an eGFR ranging between 88 and 89 mL/min/1.73 m².

**Figure 3:**
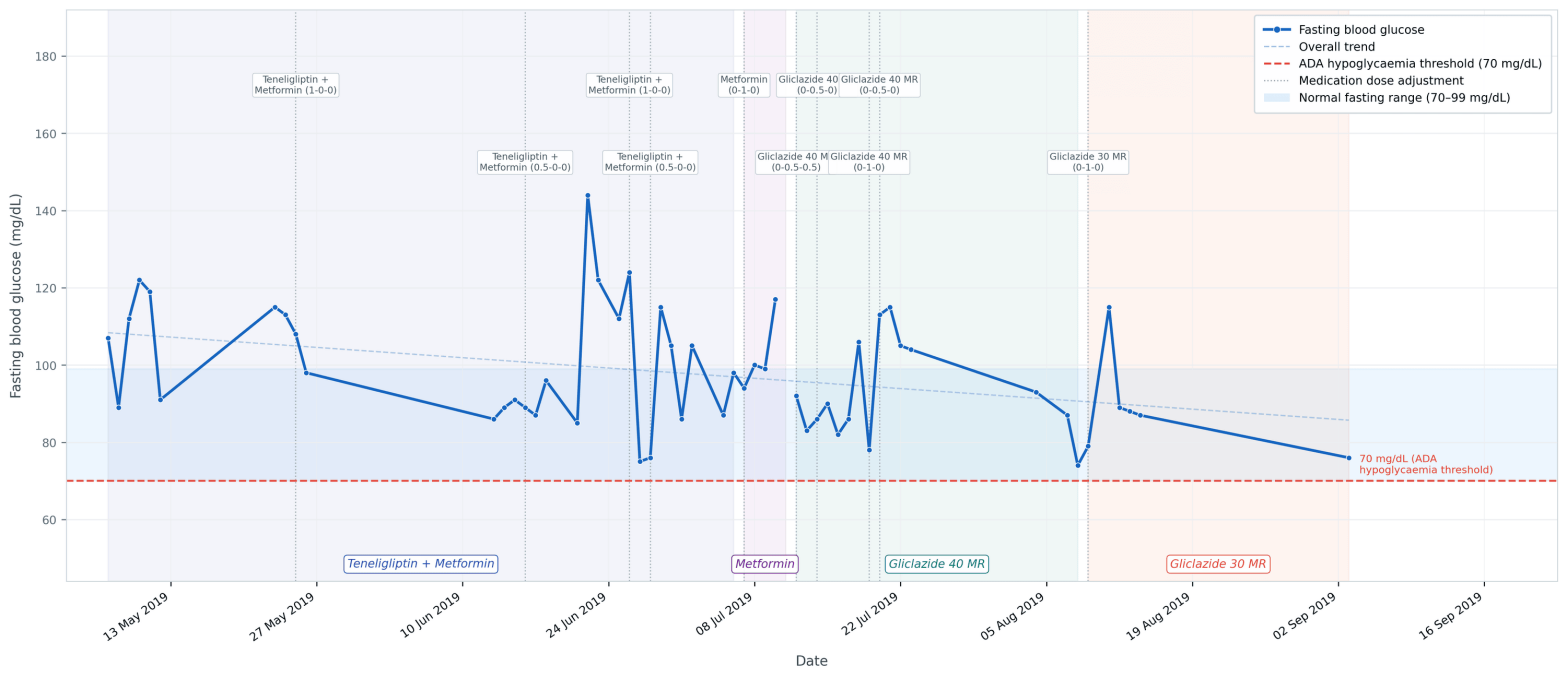
Daily fasting blood glucose levels following insulin discontinuation (May-September 2019) Vertical dotted lines indicate medication dose adjustments. Shaded bands denote medication phases. The dashed blue line indicates the overall downward trend in fasting glucose. The red dashed line marks the ADA Level 1 hypoglycemia threshold (70 mg/dL) [26]. All fasting values remained above this threshold throughout the monitoring period, with no rescue intervention required. Image created using Python (matplotlib library; https://matplotlib.org/). ADA, American Diabetes Association

Adjustments to oral medications were made based on ongoing glycemic and clinical evaluations, with each change linked to the corresponding biochemical outcomes. By May 1, 2019, one month into the intervention, daily glucose monitoring confirmed fasting glucose values within an acceptable range throughout insulin withdrawal, with a downward trend consistent with improving endogenous insulin sensitivity. This was reflected in a reduction in fasting insulin from 14.5 μU/mL at baseline to 4.8 μU/mL at three months, with HbA1c remaining stable at 7.0%. The daily glucose trajectory during this critical transition period is illustrated in Figure 3, confirming glycemic stability and the absence of hypoglycemic events throughout the insulin withdrawal period [26]. Metformin (750 mg/day) was continued during the first 12 months, a period during which HbA1c progressively declined from 7.0% to 6.6%. At 12 months, teneligliptin (20 mg/day) was added for further optimization, corresponding to a continued improvement in HbA1c to 6.5% by 15 months. Metformin was subsequently discontinued, and the regimen was simplified to gliclazide 60 mg twice daily. This simplification did not adversely affect glycemic control, with HbA1c remaining stable at 6.5% at 19 months and declining to 6.1% at 56 months. This sequential, biochemically guided approach to medication management enabled safe deprescribing while maintaining and progressively improving metabolic outcomes during the follow-up period.

To understand the physiological mechanism underlying this sustained insulin independence, endogenous insulin production was assessed at the 77-month follow-up visit. The results revealed a fasting insulin level of 4.2 μU/mL and a C-peptide level of 1.36 ng/mL. These values suggest that the sustained discontinuation of exogenous insulin was likely supported by a combination of improved insulin sensitivity and preserved endogenous insulin secretion from residual β-cell function.

## Discussion

This case describes successful insulin discontinuation in a 78-year-old patient with a 40-year history of T2D following ILI, with several factors likely contributing to this outcome. The dietary, physical activity, and behavioral components of the intervention played distinct but complementary roles.

Whole-food, plant-based diets likely support insulin sensitivity through multiple mechanisms. A high fiber content and elimination of saturated fats may address insulin resistance pathophysiology, whereas antioxidants and micronutrients support metabolic function [27,28]. The exclusion of dairy products has been associated with reduced systemic inflammation and ectopic fat burden in previous studies [29], and the observed improvement in the QUICKI in this patient reflects this enhanced sensitivity. The structured exercise program, combined with dietary modification, was associated with significant weight loss (9.4 kg) and likely contributed to improved glucose utilization. Enhanced muscle mass, increasing from 26.6% to 28% at six months, may have further supported metabolic capacity and insulin sensitivity, potentially contributing to sustained glycemic stability (HbA1c 6.6% at 77 months), as muscles may have become more efficient at utilizing available glucose following insulin withdrawal [8]. Behavioral support addressing stress management and adherence barriers is equally important for long-term sustainability, as comprehensive programs that incorporate mental and behavioral components are associated with higher engagement and sustained outcomes [30].

The physiological basis for insulin discontinuation in this patient was supported by the biochemical findings at the 77-month follow-up. Fasting insulin (4.2 μU/mL) and C-peptide (1.36 ng/mL) levels confirmed preserved endogenous insulin secretion, indicating a meaningful residual β-cell reserve. Insulin discontinuation in individuals with long-standing T2D is physiologically plausible when ILIs significantly improve metabolic function. Caloric restriction may reverse glucotoxicity, a condition in which chronic hyperglycemia impairs β-cell function [31], while significant weight loss reduces visceral adiposity, improves hepatic and skeletal muscle insulin sensitivity, and counteracts age-related insulin resistance [32]. Together, these mechanisms may substantially reduce exogenous insulin requirements, supporting gradual tapering in selected patients. The continued requirement for gliclazide in this case likely reflects partial rather than complete β-cell recovery, consistent with the hypothesis that both improved insulin sensitivity and residual β-cell function are necessary contributors to the sustained insulin-free status achieved.

The role of gliclazide warrants further investigation. Sulfonylureas are often introduced during the early stages of T2D but may also be appropriate in the later stages of the disease when residual β-cell function persists [33]. In this case, the improvement in insulin sensitivity observed following ILI was associated with reduced exogenous insulin requirements, supporting gradual tapering and eventual discontinuation of insulin therapy. The ongoing need for gliclazide underscores that the outcome represents insulin independence rather than complete pharmacological remission, and that oral pharmacotherapy remains an important component of glycemic management throughout the follow-up period.

Further, these outcomes must be interpreted within the framework of geriatric diabetes management. While the reduction in HbA1c from 7.0% to 6.6% at 77 months represents a modest absolute decrease, with the lowest value of 6.1% recorded at 56 months, its clinical significance is underscored by two key considerations. First, this level of glycemic control was achieved following the complete withdrawal of 31 years of exogenous insulin therapy, substantially reducing the risk of hypoglycemia, which is particularly critical in older adult patients. Second, the sustained glycemic stability (HbA1c consistently ≤6.6% over 77 months) and preservation of renal function (eGFR 88-89 mL/min/1.73 m² at the last available measurement) suggest that lifestyle modification may achieve clinically meaningful outcomes aligned with the principles of avoiding over-treatment and minimizing polypharmacy in elderly patients. These findings support the feasibility of deprescribing insulin in carefully selected older adults with preserved β-cell function and adequate functional status.

Limitations and interpretation

As this is a single-case report, the findings presented here cannot establish causality or be generalized to all patients with long-standing T2D. The observed outcomes, particularly the successful discontinuation of insulin, were likely attributable to a combination of factors, including ILI, structured medication de-prescription guided by daily glucose monitoring, and continued use of oral pharmacotherapy (gliclazide). The patient's relatively good baseline glycemic control and preserved renal function may not represent all older adults with diabetes, particularly those with more advanced disease. His preserved residual β-cell function, confirmed by a C-peptide level of 1.36 ng/mL, was likely an important contributor to the sustained insulin-free status. Future studies should incorporate standardized frailty assessments and formal patient selection criteria to better define which older adults with long-standing T2D may benefit from similar interventions.

## Conclusions

This case demonstrates that sustained insulin discontinuation is achievable in a 78-year-old patient with a 40-year history of T2D and 31 years of insulin dependence in the context of a structured ILI. Over 77 months of follow-up, the patient maintained glycemic control without exogenous insulin, supported by ongoing oral therapy with gliclazide and adherence to lifestyle modifications. The absence of hypoglycemic events and preservation of renal function highlight the safety of this approach in the geriatric population when implemented with structured clinical supervision. These findings are hypothesis-generating and suggest that insulin discontinuation may be feasible in carefully selected older adults with preserved residual β-cell function and adequate functional status. However, given the single-case design, concurrent introduction of multiple intervention components, and continued use of oral pharmacotherapy, causality cannot be definitively attributed to any single element of the intervention. Further research is needed to establish generalizability, define optimal patient selection criteria, and evaluate the long-term safety and efficacy of this approach in a broader population.
